# A Novel Topology Optimization Protocol Based on an Improved Crow Search Algorithm for the Perception Layer of the Internet of Things

**DOI:** 10.3390/biomimetics8020165

**Published:** 2023-04-19

**Authors:** Yang Bai, Li Cao, Binhe Chen, Yaodan Chen, Yinggao Yue

**Affiliations:** 1 School of Intelligent Manufacturing and Electronic Engineering, Wenzhou University of Technology, Wenzhou 325035, China; 2 Intelligent Information Systems Institute, Wenzhou University, Wenzhou 325035, China

**Keywords:** Internet of Things, wireless sensor networks, cluster routing, crow search algorithm, Cauchy mutation, topology optimization

## Abstract

In wireless sensor networks, each sensor node has a finite amount of energy to expend. The clustering method is an efficient way to deal with the imbalance in node energy consumption. A topology optimization technique for wireless sensor networks based on the Cauchy variation optimization crow search algorithm (CM-CSA) is suggested to address the issues of rapid energy consumption, short life cycles, and unstable topology in wireless sensor networks. At the same time, a clustering approach for wireless sensor networks based on the enhanced Cauchy mutation crow search algorithm is developed to address the issue of the crow algorithm’s sluggish convergence speed and ease of falling into the local optimum. It utilizes the Cauchy mutation to improve the population’s variety and prevent settling for the local optimum, as well as to broaden the range of variation and the capacity to carry out global searches. When the leader realizes he is being followed, the discriminative probability is introduced to improve the current person’s location update approach. According to the simulation findings, the suggested CM-CSA algorithm decreases the network’s average energy consumption by 66.7%, 50%, and 33.3% and enhances its connectivity performance by 52.9%, 37.6%, and 23.5% when compared to the PSO algorithm, AFSA method, and basic CSA algorithm.

## 1. Introduction

Wireless sensor networks (WSNs), a crucial component of the Internet of Things, are designed to gather data within a predetermined range in order to monitor the coverage area. A vast number of inexpensive microsensor nodes placed across the monitoring region make up wireless sensor networks, which are multi-hop self-organizing network systems [1]. The sensor nodes are well suited for Internet of Things environments because of their compact size and affordable price, and they are utilized extensively in a variety of sectors including business, agriculture, transportation, military, security, healthcare, space detection, and home and office environments [2,3]. The key features of wireless sensor networks for the perception layer of the Internet of Things are restricted computation and storage energy, limited communication capabilities, and limited power supply energy for sensor nodes. These qualities also serve as the primary constraints on routing protocols [4]. Therefore, it is crucial to use wireless sensor networks in an energy-efficient manner. A major obstacle in the study of wireless sensor networks is how to increase the network’s energy efficiency. The energy efficiency of WSNs may be significantly increased by the adoption of effective routing algorithms [5]. It is challenging to supply energy to the sensor from the outside world due to its tiny size, limited energy, and environmental limitations. They have to keep working until all of the energy is used up. The concept of clustering is frequently utilized in network routing protocols to decrease the quantity of data transmission and node energy consumption. A more effective method that better fits the features of wireless sensor networks is thought to be the clustered routing algorithm.

According to the characteristics of the topology of WSNs, routing protocols may be separated into flat routing protocols and cluster routing protocols. The plane routing protocol’s nodes all have the same structure and purpose, but because resource optimization management cannot be carried out without a central management node, it is only appropriate for small-scale networks [6,7]. The network is divided into numerous clusters using the clustering routing protocol. Each cluster has a cluster head node that coordinates communications with the other cluster members and combines and broadcasts received data to the base station. The cluster head must receive, combine, and transfer data collected by cluster member nodes throughout the whole network, which frequently uses more energy [8]. A cluster head node will fail early if there are not enough cluster heads, and if each cluster head is receiving and transmitting an excessive quantity of data. A network’s lifespan will be shortened by an excessive number of cluster heads, which will increase their energy consumption when connecting to the base station. Therefore, another crucial aspect of network energy usage is the number of cluster heads [9]. It is crucial to pick the right number of clusters to minimize energy usage and increase the network’s lifetime.

*A*. *MOTIVATIONS*

A crucial piece of technology needed to address the issue of WSNs is the clustering network structure. Many new clustering algorithms have been presented by academics both domestically and overseas. In this paper, a previously developed, effective clustering routing protocol is presented [10]: the clustering data-collecting approach. In order to save energy and prolong the lifespan of the network, the protocol randomly chooses cluster heads in a loop and uniformly distributes the energy burden of the whole network to each node. However, the lack of consideration for elements such as the node’s location and remaining energy results in an unequal distribution of cluster heads. In the clustering network structure made up of the particle swarm optimization technique, P. C. Srinivasa Rao et al. [11] determined the ideal number of clusters with the objective of reducing the actual energy consumption of the network. The network model, however, is too perfect; the communication model it uses overlooks route loss and does not account for the energy loss brought on by network nodes receiving data during transmission.

The choice of cluster head is modeled as an NP-hard issue in this research by looking at a number of variables that have an impact on the energy consumption of cluster head communication in the cluster routing protocol, and a fitness function is developed for cluster head evaluation. Additionally, the swarm intelligence optimization technique is used to optimize the number of cluster heads in order to provide a collection of cluster heads with greater energy and shorter communication distances, hence lowering network energy consumption. In order to decrease the frequency of cluster head replacements and thus lower cluster energy consumption and extend the lifespan of the network, an energy threshold is included in the network clustering stage.

*B*. *CONTRIBUTIONS*

The Cauchy mutation crow search algorithm (CM-CSA) is the foundation of the novel energy-saving and effective clustering routing protocol proposed in this study. The Cauchy mutation crow method improves on the normal CSA algorithm’s global search performance by preventing convergence towards the local optimum and increasing convergence speed and accuracy. The cluster routing protocol, which also takes into account the node’s remaining energy, its distance from the sink node, and its range, among other factors, efficiently balances the node load, lowers energy consumption, and boosts network effectiveness. The following can be used to summarize the research’s key contributions:

(1) A mathematical model was established by reviewing the existing cluster routing protocol of WSNs.
(2) A novel cluster routing protocol optimization method was developed using the enhanced crow search algorithm.
(3) The results of the simulations were utilized to verify the suggested algorithm’s efficacy and efficiency.
(4) The performance metrics of the proposed CM-CSA algorithms were compared to those of the PSO, AFSA, and basic CSA algorithms.

## 2. Related Work

The key to extending the lifespan of wireless sensor networks is addressing the issue of network energy consumption. Hierarchical routing’s clustered routing algorithm is a crucial and useful approach to addressing the issue of network energy usage. However, it is challenging to establish uniform clustering and a maximum balance of the energy consumption for wireless sensor networks. Venkataraman et al. clustered the wireless sensor network and sent the data to the sink node using the energy-optimized dynamic clustering (EODC) hierarchical routing protocol for WSNs. Until it reaches the base station, this approach communicates amongst cluster heads using the shortest way method. The network’s longevity and energy efficiency are increased in this way [12]. Su et al. suggested an energy-efficient clustering technique for wireless sensor networks based on fuzzy C-means to balance the energy consumption of nodes in energy-constrained wireless sensor networks and lengthen the life cycle of nodes. It is suggested sensor nodes be partitioned into a predetermined number of groups using an updated fuzzy C-means clustering method. This technique successfully lowers network energy use [13]. Distance and energy-constrained k-means clustering strategy (DEKCS) cluster head selection is a novel technique put out by the author of [14]. The cluster head is chosen based on the remaining battery power and the cluster’s probable cluster head’s location. To make sure that the network entirely runs out of energy before disconnecting, we dynamically adjust the remaining energy threshold specified for possible cluster heads. In [15], the author put out an interval-model-based optimization technique for determining the size of opportunistic signal clusters in wireless sensor networks. This technique significantly increases data collecting efficiency while consuming less network energy. Multi-level clusters, according to the author’s argument in [16], not only allow energy-saving data-gathering techniques, but also offer greater scalability for large-scale sensor network data collection. This technique successfully lowers network energy usage and increases the network’s lifespan. The author of [17] suggested an adaptive neuro-fuzzy clustering method that chooses candidate cluster heads based on three input parameters: residual energy, base station distance, and neighborhood distance. Next, the cluster heads compete using the deer hunting optimization (DHO) method, and then choose the top cluster heads. The fitness function is derived by DHO-based clustering technique using residual energy, distance to base station, node degree, node centrality, and connection quality. Utilizing this technique substantially lowers network energy usage.

With the development of swarm intelligence optimization algorithms in recent years, clustering data collecting of wireless sensor networks has become more and more common. Numerous metaheuristic techniques are utilized to address the wireless sensor network clustering problem since it is an NP-hard problem. By using the particle swarm optimization algorithm (EC-PSO), Wang et al. devised an energy center search clustering approach that prevents the creation of energy holes and identifies energy centers for the selection of cluster heads [18]. To provide safe data transmission by identifying and following the optimum path, Prithi et al. used the deterministic finite automata (DFA) and particle swarm optimization method (PSO). To foster the network’s dynamic properties, a brand-new learning dynamic deterministic finite automaton (LD2FA) is suggested. The entire performance of the sensor network may be improved by routing through the best way, and this is measured by metrics such as energy consumption, throughput, and network lifespan [19]. The nodes are separated into many clusters, one of which acts as a cluster head (CHs) to receive and consolidate the received information, in order to lower the overall network’s energy usage. An NP-hard task is the selection of CHs in the best way. In [20], the author developed an enhanced artificial fish school algorithm (AFSA)-based CH selection method that greatly lowers network energy consumption and boosts network effectiveness. In [21], a hybrid optimization technique developed by the author, known as the hybrid particle swarm optimization–grey wolf optimization algorithm (PSO-GWO), efficiently uses energy and transmits data in a secure manner along an improved path. The authors of [22] suggested an enhanced chaotic whale-based optimization algorithm (OBC-WOA) in order to increase the effectiveness and dependability of clustered data collecting. The OBC-WOA algorithm makes use of an opposition-based technology to increase its effectiveness. In terms of energy conservation, network survival, data packet transfer, and consumption, the algorithm performs quite well.

The three primary arguments may be summed up based on the aforementioned papers. (1) The direction of the study is largely biased. If the clustering approach under consideration is more complex, many researchers studying hierarchical cluster routing will overlook the routing between clusters. On the other hand, basic and underperforming clustering algorithms are employed while researching sophisticated inter-cluster routing. (2) There is a lot of unpredictability in clustering or choosing cluster heads. The thresholds of various node weight factors are often determined by current clustering algorithms to select potential cluster heads. The cluster head is then selected with equal probability by each node randomly generating a random number. (3) The clustering approach has a bias. The notion of non-uniform clustering, graph theory, perceptions of energy, and geographic location are only a few of the foundations upon which many contemporary studies on clustering tactics are built. These tactics should perform better because they simply take into account one factor.

One effective optimization technique is the crow search algorithm. The aforementioned cluster routing issues may be resolved successfully by using it to tackle the cluster optimization problem. Based on the study presented above, this paper develops a Cauchy mutation crow search (CM-CSA)-based clustering routing method for wireless sensor networks. The crow search technique is enhanced with Cauchy mutation to help it converge quickly and optimize precisely. The suggested algorithm improves the performance of WSNs by successfully balancing the load, the distribution of network node cluster heads, and the network energy consumption.

## 3. Mathematical Model

There is an ideal range for the number of clusters; however, most clustering routing methods assume a fixed ratio of cluster heads. If there are not enough cluster heads, certain member nodes will be too far from the cluster head, requiring more energy for transmission. Certain nodes in the cluster will not be able to send data because they are too far from the cluster head. Data loss is brought on by the cluster head. In contrast, if there are too many cluster heads, they will need more energy to communicate with the base station or other cluster members. In order to determine the ideal number of cluster heads, this research examines the effect of the number of cluster heads on the overall network usage. The base station is where the clustering procedure and the estimation of the ideal number of cluster heads are both finished.

First, we establish a clustering mathematical model of WSNs based on energy consumption. The monitoring area is a square of length *M*, and the total number of nodes is *N*, the cluster head nodes are *K*, and there are *N*/*K*-1 cluster member nodes. The energy consumption generated by the data fusion is *E_CH_*, and then the information is transmitted to the sink, and the distance between the cluster head and the base station is *d_ChtoBS_*. The energy consumption of cluster head nodes mainly includes receiving data sent by the sensor nodes and forwarding data to the sink. The energy consumption *E_NEN_* of cluster member nodes includes collecting data and forwarding data to the cluster head.

The ordinary nodes and the sensor nodes in the sensing area obey the Poisson distribution, the area of the cluster is *S = M*^2^/*k*, and the density function of the nodes in the monitoring area is ρ(x,y), which is calculated as the following Formula (1).

(1) $$\rho (x,y)=\left\{ \begin{array}{cc} k/M^{2} & ,\;(x,y)\in S\\ 0 & ,\;(x,y)\notin S \end{array}\right.$$

The cluster head node’s energy use when receiving data is inversely proportional to the distance. The square of the distance between the cluster head node and the cluster member nodes’ mathematical expectation value is computed as

(2) $$E[d_{toCH}{}^{2}]=E[x^{2}+y^{2}]=M^{2}/2k\pi$$

The formula for calculating the sum of energy consumption of all nodes in the entire wireless sensor network is

(3) $$E_{total}=k\times E_{cluster}=k\times E_{CH}+k\times (N/k-1)\times E_{NEN}$$

(4) $$\begin{array}{cc} E_{total} & =k\times E_{cluster}=k\times E_{CH}+k\times (N/k-1)\times E_{NEN}\\ & =k\times ((N/k-1)\times lE_{elec}+lE_{elec}+l\varepsilon_{amp}d_{CHtoBS}^{2})+\\ & \;(N-k)\times (lE_{elec}+l\varepsilon_{amp}d_{toCH}^{2})\\ & =l(2NE_{elec}-kE_{elec}+k\varepsilon_{amp}d_{CHtoBS}^{2}+(N-k)\times (\varepsilon_{amp}d_{toCH}^{2}) \end{array}$$

In Formula (4), only the value of $d_{toCH}$ is uncertain. In this calculation formula, since the position and number of cluster heads must change during each round in the data collection process, the parameter $d_{toCH}$ is calculated by calculating the mathematical expectation value. The range of each cluster area is a circular area with a radius of $M/\sqrt{\pi K}$. The formula for calculating the expected value of the square of the distance from the sensor node to the cluster head node is:

(5) $$\begin{array}{cc} E\left[d_{toCH}^{2}\right] & =\iint \left(x^{2}+y^{2}\right)\rho (x,y)dxdy=\iint r2\rho (r,\theta )rdrd\theta \\ & =\rho \int_{0}^{2\pi }d\theta \int_{0}^{M/\sqrt{K}}r^{3}dr=M^{2}/2\pi K \end{array}$$

The total energy consumption of the network can be calculated as

(6) $$E_{total}=l(2NE_{elec}-kE_{elec}+k\varepsilon_{amp}d_{CHtoBS}^{2}+(N-k)\times \varepsilon_{amp}\times M^{2}/2k\pi )$$

By substituting the calculated result of Formula (5) into Formula (4), and calculating the derivative, the calculation result of the number of cluster heads *K* can be obtained. The derivative of the number of cluster heads *K* is equal to 0, and the following calculation is obtained:

(7) $$E_{elec}-\frac{N\times M^{2}\times \varepsilon_{fs}}{\pi \times K^{2}}+\varepsilon_{amp}\times (d^{4}+d_{CHtoBS}^{4})=0$$

Regarding the calculation of the optimal number of clusters *K* in Formula (7), the first derivative of *K* can be calculated to obtain the calculation result of the optimal number of clusters *K.*

(8) $$f_{1}=K=M\times \sqrt{\frac{N\times \varepsilon_{fs}}{\pi }}\times \sqrt{\frac{1}{E_{elec}+\varepsilon_{amp}\times (d^{4}+d_{CHtoBS}^{4})}}$$

It can be seen from the calculation result of Formula (8) that the calculation result of the optimal cluster number *K* value is related to the number of all sensor nodes *N* in the monitoring area. This is the objective function *f*_1_.

The objective function *f*_2_ is the communication cost function between cluster head and base station. Since the network is in the stable communication stage, the cluster head needs to forward a large amount of information to the base station, so by minimizing the objective function *f*_2_, the node closer to the base station can be selected as the cluster head to reduce the communication cost between clusters, where *f*_2_ is

(9) $$f_{2}=\frac{1}{K}\sum_{j=1}^{K}d(CH_{j},BS)$$

where $d(CH_{j},BS)$ is the distance between the cluster head *CH_j_* and the base station, and the parameter *K* is the number of cluster heads in objective function 1.

The objective function *f*_3_ is the residual energy function of the cluster head. The cluster head needs more energy to collect, fuse, and forward the information of the member nodes in the cluster in the network. By minimizing *f*_3_, the node with the highest residual energy becomes the cluster head. The expression of *f*_3_ is as follows:

(10) $$f_{3}=\frac{1}{\sum_{j=1}^{K}E_{cur}(CH_{j})}$$

wherein $E_{cur}(CH_{j})$ is the current residual energy of the cluster head *CH_j_*.

According to the above description, the optimization of wireless sensor network topology can be solved by minimizing the *fitness* function. The *fitness* expression is as follows:

(11) $$fitness=\alpha f_{1}+\beta f_{2}+\gamma f_{3}$$

where *α*, *β*, and *γ* are the weight coefficients of *f*_1_, *f*_2_, and *f*_3_, and *α* + *β* + *γ* = 1. The number of cluster heads and the choice of cluster heads in the wireless sensor network topology optimization issue are both NP-hard to solve. The swarm intelligence optimization method may solve the problem by utilizing the fitness as the fitness function and the least value as the ideal value.

## 4. Crow Search Algorithm

Askarzadeh’s crow search algorithm (CSA), a novel metaheuristic algorithm that draws inspiration from nature as well, was put out in 2016 [23]. The algorithm’s central thesis is that the crow, a social bird, has the capacity to travel, store, and discover food knowledge, as well as a sophisticated brain to guard against other companions stealing its food [24,25]. We discovered via the examination of the CSA that it may be utilized to address the discrete space search issue, particularly the issue of feature selection. The crow method is simple to use and quickly converges since it only requires two parameters: flight length *fl* and awareness probability *AP*. As a result, the crow algorithm offers some potential for application research in a variety of industries and is more competitive than other intelligent optimization algorithms [26].

A reasonable number of iterations, *iter*, can be set; $X^{i,iter}=x_{1},x_{2},x_{3},\cdots ,x_{n}$ is the crow coordinates in the *d*-dimensional space, the crow population is *n*, $Memory,(m^{i,iter}=m_{1},m_{2},m_{3},\cdots ,m_{n})$ is the storage point of hidden food, and the storage is used to represent the current best coordinates obtained by the crow individual *i*.

If the crow individual *i* is the chasing individual and *j* is the chased individual, when the crow individual *j* is not aware of the existence of the stealer, this situation is attributed to state *A*; that is, the crow individual *j* has not found the tracking of the crow individual *i*. Then, in this state, crow *i* will generate a new coordinate, which can be expressed as [27]

(12) $$X^{i,iter+1}=X^{i,iter}+r_{i}\times fl^{i,iter}\times (m^{j,iter}-X^{i,iter})$$

where the parameter $\;r_{i}$ represents a random number within [0, 1], and $fl^{i,iter}$ is the flight distance of crow *i* in the *iter* iteration, also called flight step length. The size of the flight distance has a different effect on the search ability of the algorithm. A smaller *fl* value is helpful for individual crows to perform a local search, while a larger *fl* value can guide the crows to perform a global search [28].

In this state, the crow individual *j* finds the tracking of the crow individual *i*. In order to prevent the crow *i* from discovering the coordinates of its hidden food, it will mislead crow *i* and disturb its audiovisual sensory information, so that the crow *i* will fall to a random coordinate in the search space. Therefore, these two different chasing states can be summarized as

(13) $$X^{i,iter+1}=\left\{ \begin{array}{cc} X^{i,iter}+r_{i}\times fl^{i,iter}\times (m^{j,iter}-X^{i,iter}), & r_{i}\geq AP^{j,iter\;}\;\\ a\;\mathrm{random}\;\mathrm{position} & \mathrm{otherwise} \end{array}\right.$$

The parameter $AP^{j,iter\;}$ represents the perception probability *AP* of the crow individual *j*. In the crow search algorithm, the probability of consciousness controls the convergence and diversity of the algorithm. Different *AP* values play different roles. When the *AP* value is reduced, the algorithm tends to perform a local search. A smaller *AP* value can increase the convergence of the algorithm. When the value of *AP* increases, the crow search algorithm will tend to search globally, and the probability of searching nearby areas to obtain a solution will decrease, but the diversity of the algorithm can be increased [29].

## 5. Cauchy Mutation Crow Search Algorithm

The 2016 metaheuristic method known as the “crow search algorithm” offers the benefits of a straightforward structure and straightforward implementation. By mimicking crow foraging behavior, the algorithm is proposed. Due to a single population and unequal distribution at the start of the iteration, the procedure is prone to local optimality and has poor optimization accuracy. This research suggests an enhanced crow search algorithm (CM-CSA) based on Cauchy mutation as a solution to these issues. The problem of the algorithm slipping into the local optimal is successfully resolved, and the precision of the solution is increased by the use of the Cauchy mutation operator to enhance the global optimal individual. The global optimal individual is utilized in the position update strategy to direct the crow’s location without being detected by the guide, and the next position is acquired, thus reducing the algorithm’s blindness and accelerating convergence. The following three areas are where the algorithm is mostly being improved.

(1) Global optimum individual’s Cauchy mutation method

The ideal individuals in the crow population are subjected to mutation operations using the mutation method to produce people with significant global search skills. This causes the algorithm to depart from the local optimum and produce these individuals. It can be seen that the Cauchy variation has a stronger perturbation ability and can obtain a better optimization range because the standard Cauchy distribution’s zero-point peak is lower than the standard Gaussian distribution’s and the downward trend on either side of the zero point is slower. The Cauchy mutation operator is introduced to improve the global optimal individual $g_{best}$, which effectively solves the problem of the algorithm falling into the local optimum and improves the accuracy of the solution. The Cauchy variation formula is as follows:

(14) $$X_{i}^{\prime }=\left\{ \begin{array}{cc} X_{i}\times Cauchy(0,1), & rand(0,1)\leq p\\ X_{i}, & others \end{array}\right.$$

where Cauchy(0,1) is the standard Cauchy distribution function, rand(0,1) is a random number uniformly distributed between 0 and 1, and the parameter *p* is the probability of random variation.

According to Formula (14), the Cauchy mutation operator is introduced to modify the global optimal individual $g_{best}$ to obtain the Cauchy global optimal individual. The specific operations are as follows:

(15) $$Cauchyg_{best}=\left\{ \begin{array}{cc} g_{best}\times Cauchy(0,1), & rand(0,1)\leq p\\ g_{best}, & others \end{array}\right.$$

(2) New adaptive step size

The step size *fl* in the crow search algorithm affects the convergence accuracy and speed of the algorithm. A smaller step size has more detailed local search ability, and a larger step size can exert larger global search ability. In each iteration, the step length is adaptively adjusted and changed according to the distance $D_{i}^{t}$ between the leader *j* and the current individual. The step length of each individual in each iteration changes as follows:

(16) $$fl^{i,iter}=D_{i}^{t}\times \lambda$$

where the parameter $D_{i}^{t}$ represents the distance between the leader *j* and the current individual, and the parameter λ is the scaling factor. The proposed algorithm controls the step length change within a reasonable range according to the set threshold, thereby adjusting the value of the step length. It can be seen from the expression of the parameter $D_{i}^{t}$ that if the parameter $D_{i}^{t}$ is larger, the current position is farther from the leader and the step size is larger, and the proposed algorithm reflects the global search performance. If the parameter $D_{i}^{t}$ is smaller, the distance is closer. The step size is smaller right now. The step size is automatically lowered and the local area is played around the better person as the gap between the present individual and the ideal individual steadily shrinks in the latter stages of optimization. The ability to manage population growth and variety is provided via the search function.

(3) New location update strategy

Assume that all crow individuals can obtain the global optimal position of $g_{best}$ according to their own thief experience and their own memory matrix. When performing the next position update, assume that crow *i* randomly selects crow *j* for tracking. If crow *j* does not know that crow *i* is tracking it, then crow *i* is close to the best position for crow *j* to hide food, but the position of *j* is not necessarily good. Therefore, the global optimal individual $g_{best}$ is introduced to guide the position update operation, and crow *i* can be based on the optimal position. The direction of the following step forward is decided using both the location information and the location information of crow *j*. The issue of the algorithm entering the local optimum is resolved by using the global optimum after Cauchy mutation as guidance.

(17) $$X^{i,iter+1}=g_{best}\times Cauchy+r_{i}\times fl^{i,iter}\times (m_{j}^{{}^{i,iter}}-X^{i,iter})$$

**Figure 1:**
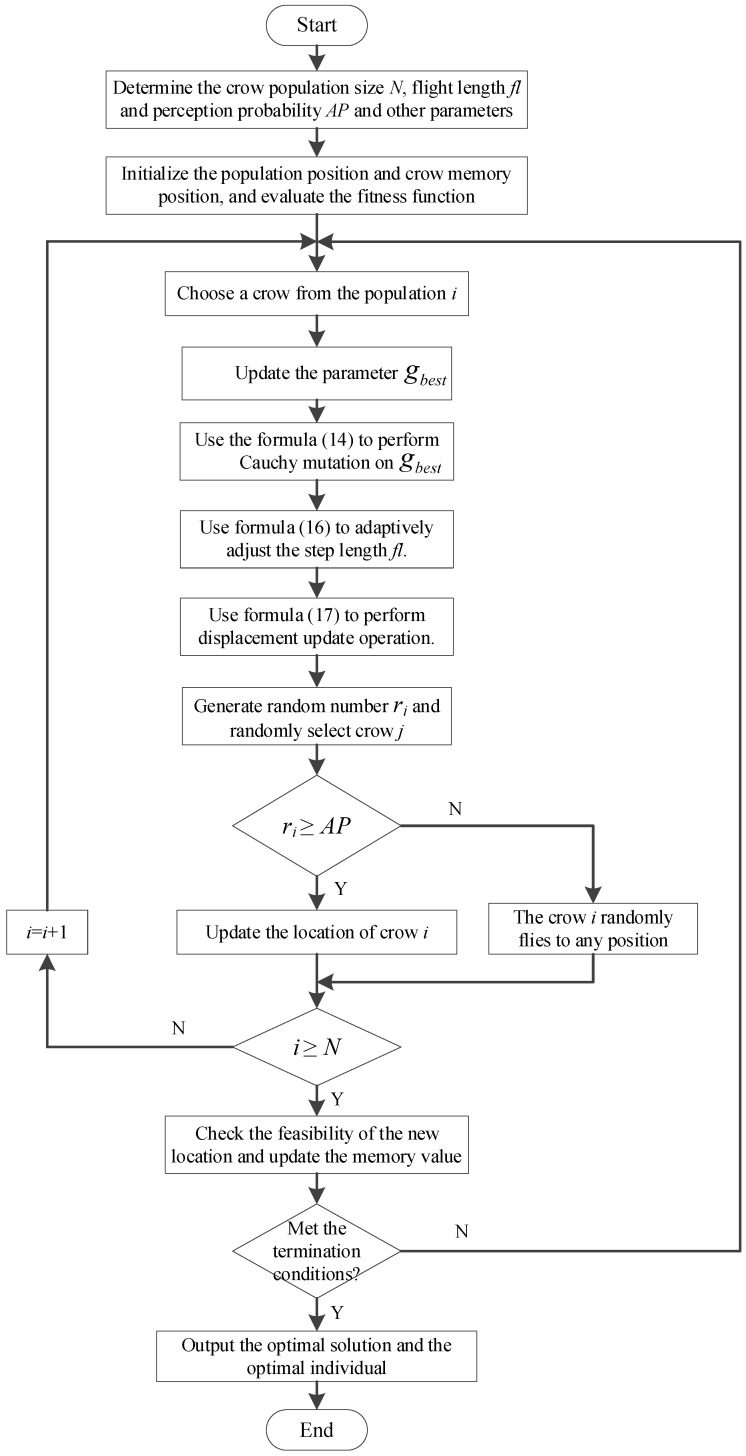
The workflow of the CM-CSA algorithm.

The workflow of the CM-CSA algorithm is shown in Figure 1.

Table 1 displays the procedures for implementing the CM-CSA method suggested in this work.

## 6. Application of CM-CSA Algorithm in the Clustering of WSNs

The WSN optimum clustering number clustering application implementation stages are broken down into the following four parts based on the CM-CSA method.

(1) Initially clustering

*N* sensor nodes are inadvertently distributed in a monitoring region during the network configuration phase. All nodes communicate their own energy and location data to the base station during the first clustering phase. The base station collects and stores the position and energy information for each network node. The cluster head will be chosen from the candidate cluster heads by calculating the average energy based on the data collected from all nodes, setting it as the energy threshold, and selecting the node with the most energy remaining after the energy threshold. The base station then chooses any cluster head from the candidate cluster heads as a crow search operator when the clustering algorithm is conducted, according to the prior optimal number of cluster heads.

(2) Determining the ideal number of WSN clusters using the CM-CSA algorithm

Equation (8) provides the calculation method for determining the optimal number of cluster heads K given the goal function. The next step is to utilize the CM-CSA algorithm global search strategy to compute the ideal number of clusters *k* based on the optimal number of cluster heads.

The calculation steps of the CM-CSA algorithm are shown in Table 1. The ideal cluster head number K of the objective function equals the estimated optimal individual location of the crow, and clustering is carried out.

(3) Stable operation stage

The cluster head will establish the member nodes’ working timeslots, after which each node in the cluster will awaken at its own time, watch the monitoring area, gather data, and report it to the cluster head. After gathering data from cluster nodes, the cluster head performs data fusion and other processing procedures before transmitting data in accordance with inter-cluster routing. The next round of clustering is carried out when the residual energy of a particular percentage of cluster head nodes is lower than the threshold.

(4) Algorithm evaluation

This algorithm’s primary method of achieving energy savings and balancing energy consumption is through the implementation of an upgraded CM-CSA algorithm to optimize clustering. The upgraded crow search algorithm can swiftly and accurately search for the optimal solution while successfully preventing the algorithm from entering the local optimal. The suggested clustering strategy minimizes the development of network holes, and careful consideration can enable the algorithm to balance and reduce energy consumption, increase network effectiveness, and extend network lifetime.

In Table 2, the pseudo-code for the CM-CSA-based energy-saving, effective, and dependable clustering data-gathering technique for WSNs is displayed.

## 7. Algorithm Simulation and Result Analysis

### 7.1. CEC Benchmark Function Test

The eight benchmark functions are displayed in Table 3. The performance of CM-CSA should be compared to that of the five fundamental particle swarm algorithms (PSOs), the firefly algorithm (FA), the sine cosine algorithm (SCA), the artificial fish swarm algorithm (AFSA), and the basic crow search algorithm (CSA). The test function has a size d of 30 and comprises both unimodal and multimodal functions. The simulation was conducted using the version of MATLAB 2020b, and the test was run under identical operating conditions in order to fairly verify the efficacy of the CM-CSA method. The population size is 30, there are 500 iterations, and each algorithm is executed 50 times separately under the operating system Microsoft Windows 11. The results of the test function are shown in Table 4.

The results of each method are listed in Table 4 along with the maximum value (Max), mean value (Mean), and standard deviation (Std) of the optimal solution achieved for each algorithm. Table 4 shows that, for unimodal functions, the accuracy of other algorithms in various dimensions is essentially the same, with the exception of F2 and F3. However, CM-CSA has a significantly faster convergence speed than the other five algorithms, and the average value is close to the theoretical optimal solution with the lowest standard deviation. The revised CM-CSA algorithm may successfully leap out of the local optimum and attain ideal outcomes while optimizing multimodal functions, demonstrating the viability and effectiveness of the upgraded approach. However, the CM-CSA solution’s efficiency is the greatest. Additionally, when the dimension gradually increases, the accuracy of the fundamental CSA solution declines, but the accuracy of the CM-CSA solution essentially remains same and even the accuracy of F2 continues to rise, demonstrating extraordinarily good stability. The value of CM-CSA is lower than the average value and standard deviation of multiple optimizations, demonstrating that it is substantially more stable and resilient than the other five techniques.

### 7.2. Simulation Environment Construction

The Internet of Things’ sensing layer’s wireless sensor network simulation environment is constructed as follows. There are 200 total sensor nodes distributed at random, with the initial energy set to 1 J, in a 400 × 400 m^2^ square monitoring area. A total of 10 data packets with a length of 4000 bits are transferred from the sensor node to the cluster head every minute, where they are processed via fusion before being passed to the sink. The population size of the algorithm is 30 and the maximum number of population evolutions is set to 50 in all tests. In the CSA algorithm, the flight length *fl* and the perception probability of the crow are 2 and 0.1, respectively. The PSO algorithm parameter settings are as follows: learning factor *c*_1_ = 2, *c*_2_ = 2, inertia weight factor *ω*_1_ = 0.9, *ω*_2_ = 0.4, and the maximum number of iterations is *T_max_* = 50.

### 7.3. Comparison and Analysis of Simulation Results

The performance of the WSN network is influenced by a wide range of factors. This paper, primarily from the clustering effect of WSNs, the total network energy consumption, the number of cluster head nodes, the energy consumption of cluster head nodes, the number of packets received by the sink, network load balancing, transmission delay, and network reliability experiments, reflects the superior performance of the proposed algorithm.

(1) Comparison of network clustering effect

**Figure 2:**
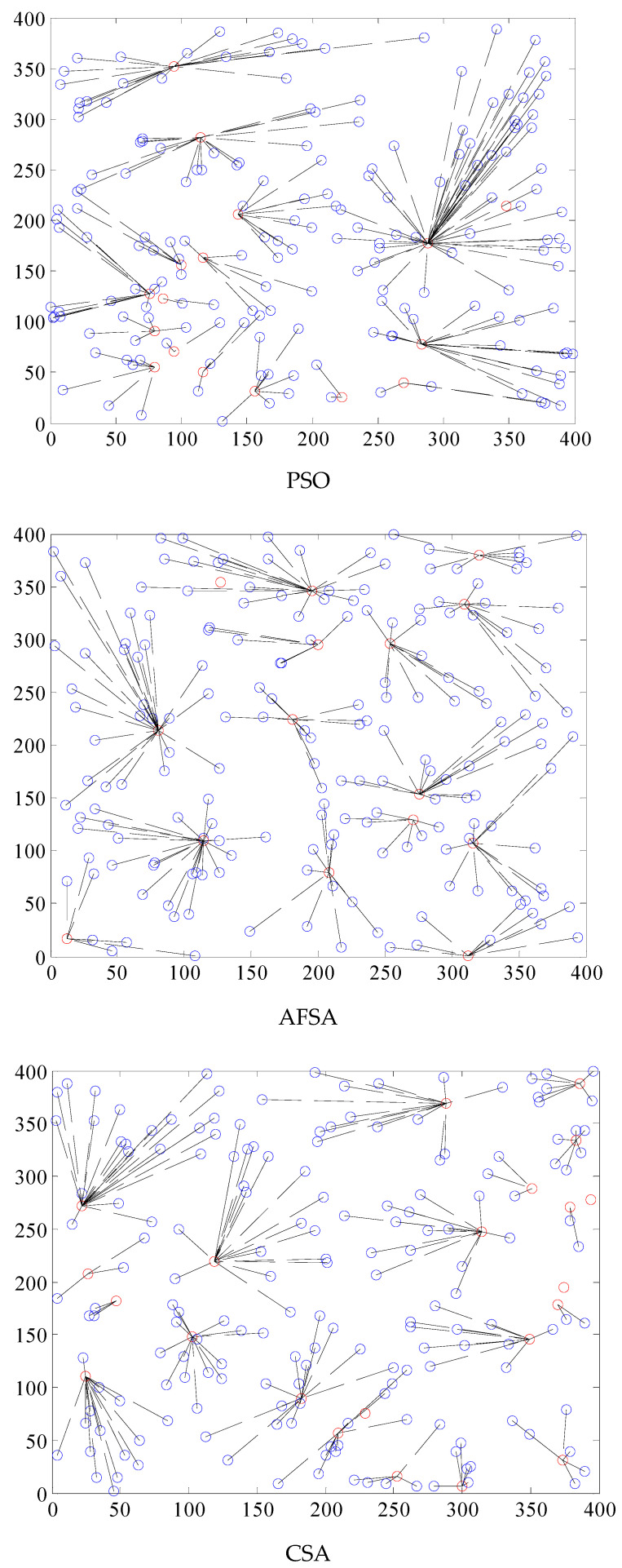

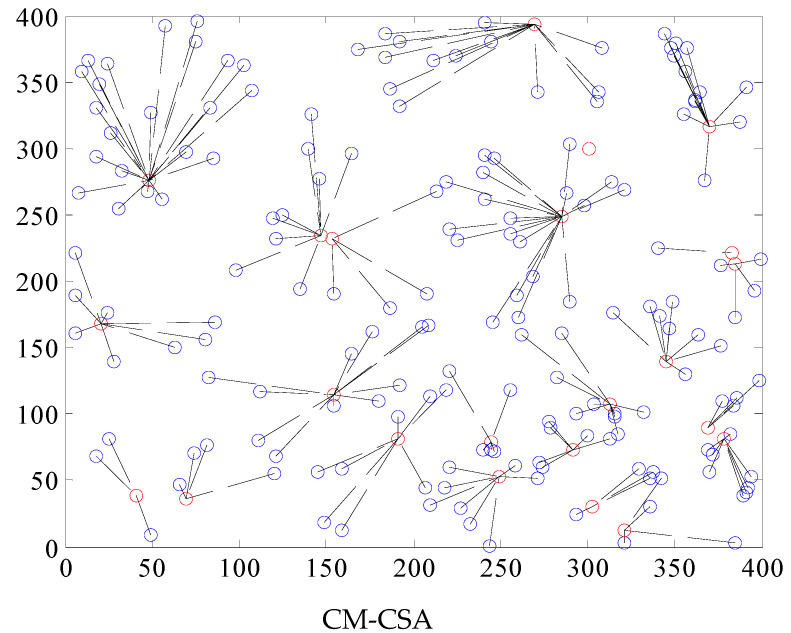
Comparison of the WSN clustering effect of four algorithms.

The energy use and longevity of the network are directly impacted by whether or not the clustering effect of the network is balanced. The nodes must use more energy to send data farther when the network is more densely packed. Multi-hop data transfer between nodes will increase the data transmission time if the clustered region is small. Figure 2 depicts the clustering effect of WSNs.

The PSO clustering impact is highly unequal, the clustering area is too big, and the data transmission latency is considerable, as can be seen from the WSN clustering effect of the four methods in Figure 1. Data transmission congestion and a higher packet loss rate can easily be brought on by the vast quantity of data that a single cluster head node receives. The AFSA method’s clustering process is rather consistent, but the distances between clusters are great, which might lead to network coverage blind patches, and the size of each cluster varies. The clustering impact on the right side of the region is very poor, the number of cluster member nodes varies substantially, and the overall clustering effect of the CSA method is rather consistent. The clustering effect is likewise weak, and there are more areas with three or four nodes. The best clustering result is provided by the CM-CSA technique suggested in this research, which also has a fair cluster head area and a somewhat uniform number of member nodes. As a result, it is clear that the CM-CSA clustering effect suggested in this study is superior to the other three techniques.

(2) Comparison of average network energy consumption

**Figure 3:**
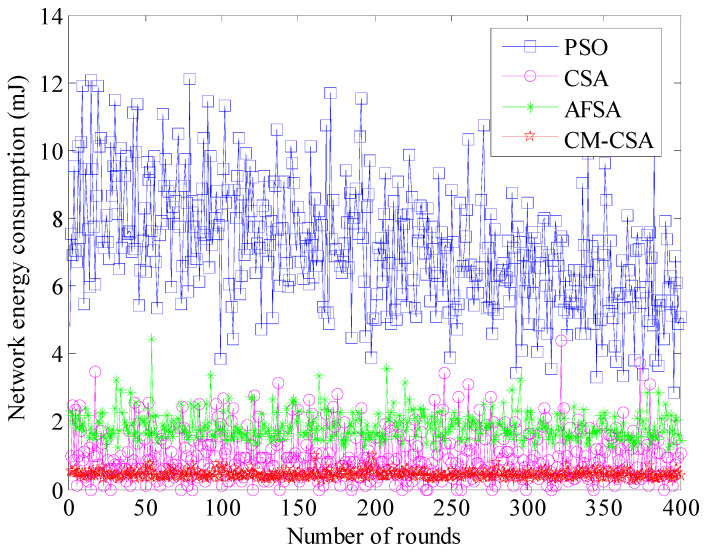
Comparison of average network energy consumption.

The most crucial performance measure for wireless sensor networks is network energy consumption. The lifespan of the network is directly correlated with its energy usage. The constant focus of wireless sensor network performance enhancement is lowering network energy consumption. Figure 3 displays the four methods’ average network energy usage.

The average total network energy used by the four methods is depicted in Figure 3 for each cycle. It is clear that the PSO algorithm uses significantly more average network energy than the other three during the course of 400 cycles. The other three methods’ average network energy use is not much different. The PSO algorithm uses an average of 6 to 10 mJ of energy every round, while the AFSA method uses an average of 2 mJ of energy per round. The proposed CM-CSA algorithm reduces the average energy consumption of the network by 66.7%, 50%, and 33.3% compared to PSO algorithm, AFSA algorithm, and basic CSA algorithm. The average energy consumption of the CSA algorithm is approximately 1.5 mJ, whereas the average energy consumption of the CM-CSA algorithm proposed in this paper is approximately 1 mJ. Overall, the approach suggested in this research uses the least amount of energy.

(3) Comparison of the number of surviving nodes in the network

**Figure 4:**
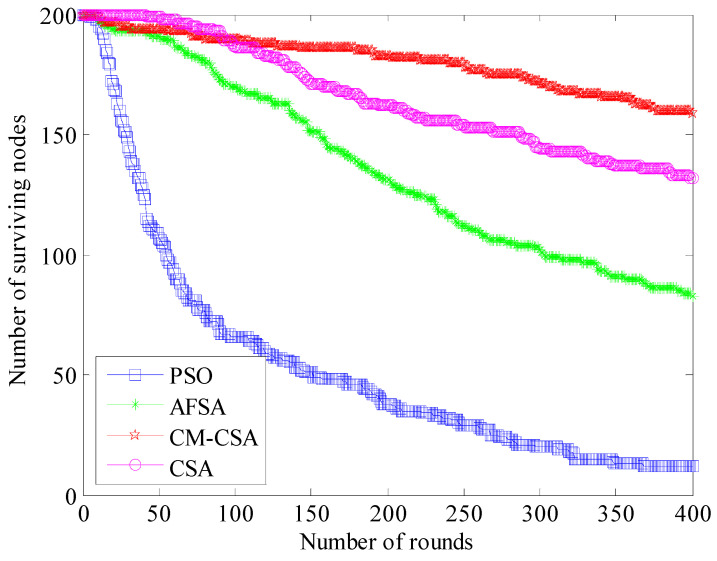
Comparison of the number of surviving nodes.

The network’s longevity may be observed directly in the number of nodes that have survived. The best network performance and the longest operating time are determined by the number of nodes that survive. Figure 4 compares how many nodes in the networks of the four algorithms are still alive.

The number of surviving nodes in the network of the four methods steadily declines as the number of simulation rounds increases, as shown in Figure 3, although the fall of the four algorithms is not uniform. The PSO algorithm’s clustering approach, which declines considerably more rapidly than the other three methods in terms of the number of surviving nodes, is one of them. Fewer than half of the remaining nodes are present after 100 cycles, which matches the simulated graph of the network’s energy usage. While the number of surviving nodes in the CSA algorithm slightly decreases, it decreases significantly in the AFSA method. The number of surviving nodes declines the least when using the CM-CSA technique suggested in this study. The PSO algorithm, AFSA algorithm, CSA algorithm, and CM-CSA algorithm each have 50, 140, 170, and 190 remaining nodes after 150 rounds, respectively. The number of surviving nodes when using the method suggested in this study rises by 73.7%, 26.3%, and 10.5%, respectively, compared to the PSO algorithm, AFSA algorithm, and CSA algorithm.

(4) Comparison of the number of cluster heads

**Figure 5:**
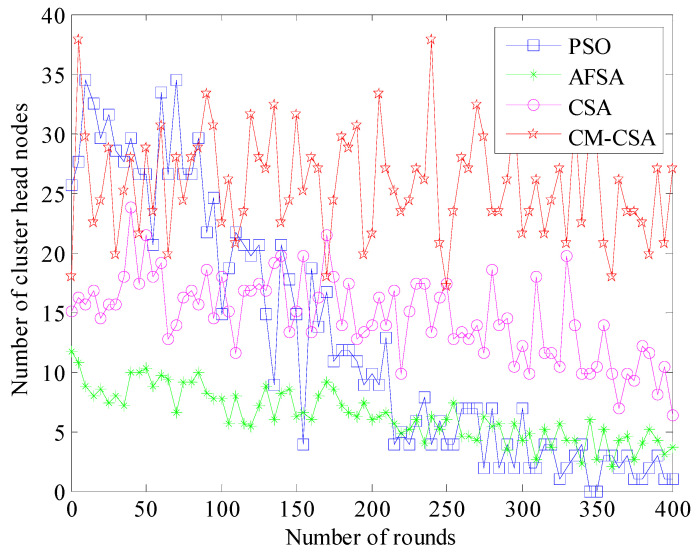
Comparison of the number of cluster head nodes.

The number of cluster head nodes represents the network’s balanced performance and is directly connected to the network’s productivity and lifespan. The network’s performance will be impacted by an excessive number of cluster head nodes. Figure 5 compares how many cluster heads each of the four strategies has.

The number of cluster head nodes, at a specific level, typically 10–15% of all nodes, can maximize the network’s performance. The performance of the network is optimized when the number of cluster heads is kept between 20 and 30 while simulating all 200 nodes in this article. Figure 4 demonstrates that only the CM-CSA technique suggested in this study maintains 20–30 cluster heads. The PSO algorithm’s cluster head count varies the most, going from 30 at the start to roughly 7 toward the end of the experiment. This is mostly due to the nodes’ uneven clustering and excessive mortality rates. The number of cluster heads evenly decreases as the simulation goes on, and the trends of the AFSA and CSA algorithms are rather comparable. However, the CSA method has more cluster heads than the AFSA algorithm and performs better; its number of cluster heads is closer to 10–15% of the total number of sensor nodes.

(5) Comparison of cluster head energy consumption

**Figure 6:**
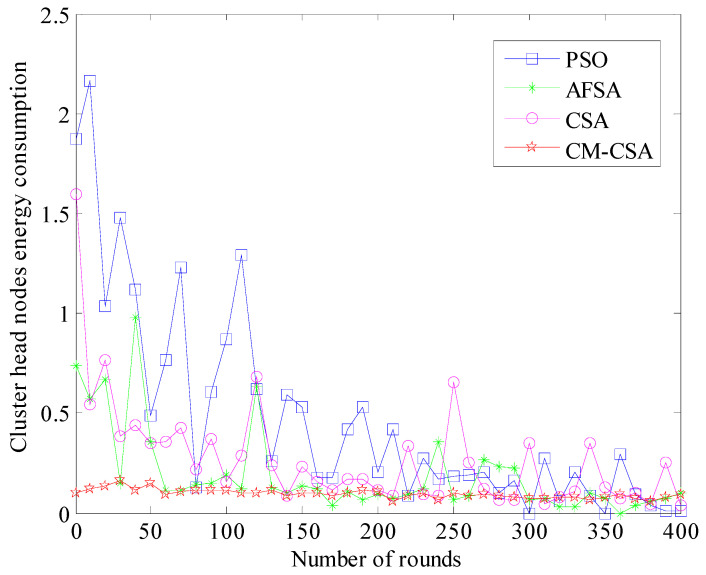
Comparison of energy consumption of cluster head nodes.

The clustering effect is reflected in the cluster head node’s energy use. The performance and clustering of the method improve with reduced total cluster head node energy consumption. The clustering effect of the method is not optimal, as seen by the cluster head node’s high energy consumption. Figure 6 displays a comparison of the four methods’ cluster head nodes’ energy usage.

Figure 6 illustrates how the energy usage of the cluster head node rapidly reduces as the number of simulation rounds rises. However, the energy consumption of the algorithm’s cluster head is rather high at the beginning of the simulation and steadily drops off as it progresses. Additionally, it can be noted that the PSO algorithm’s cluster head node has the highest energy consumption, followed by the AFSA algorithm’s cluster head node with more energy consumption and the CSA algorithm with less energy consumption. The number of cluster head nodes in Figure 4 corresponds to the energy consumption of the cluster head node of the CM-CSA algorithm that was developed in this study. The energy consumption of the network and the cluster head are both reduced due to clustering, which is balanced. Instead, there is an imbalance in the clustering, which wastes a lot of energy.

(6) The number of packets received by the sink

**Figure 7:**
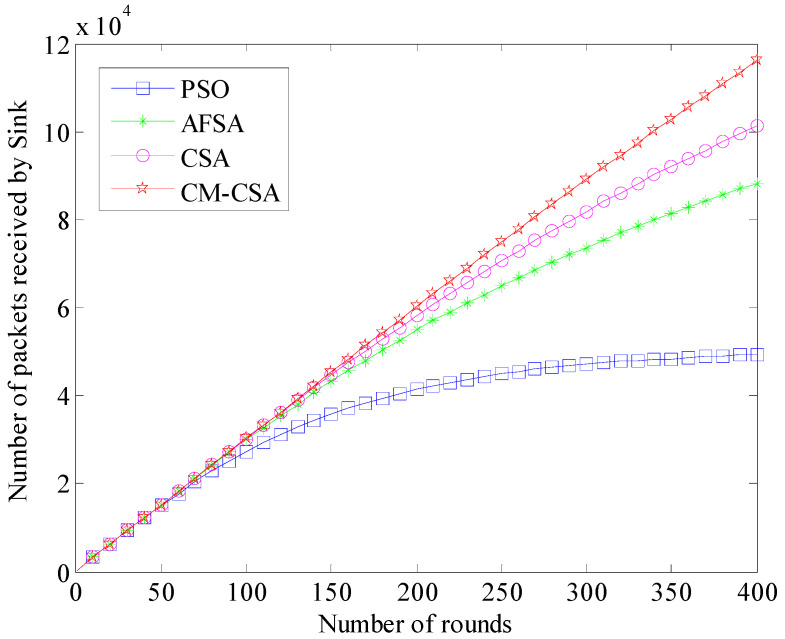
Comparison of the number of packets received by sink.

The ultimate objective of clustered data collecting is the quantity of packets received by the sink. Through multi-hop self-organization, the data gathered by regular nodes are delivered to the cluster head node, which then transmits them to the sink. The clustering algorithm is directly visible in the quantity of packets that the sink receives. The method performs better when the sink receives more packets. Figure 7 displays the number of data packets the sink received for each of the four techniques.

Figure 7 shows that as the number of simulation rounds rises, the number of sinks received by the four algorithms also rises steadily, but at different rates for each method. The PSO method has the smallest increase out of the four, and the relative differences between the other three algorithms are not very significant. This is mostly due to the PSO algorithm node dying earlier, which results in fewer data packets being received later. Overall, the AFSA algorithm’s sink receives fewer data packets, whereas the CSA algorithm’s sink receives more data packets. This study suggests that the CM-CSA algorithm’s sink receives the most data packets.

(7) Network connectivity comparison

The network connectivity rate, which is more difficult to calculate, is typically used to gauge network connection [30]. The connection rate is determined by counting the number of data transmission hops, counting the hops from the source node to the destination node, and calculating the connection rate using the nodes’ mechanism for traversing data transfer [31]. The perception node traversal technique is typically used to determine the connectedness of WSNs for a network at a specific period. Assuming a reference perception node is utilized, nodes connected from each of its first, second, and third hops should be successively determined until the number of nodes connected to the starting perception node stops growing. If we use the data transmission from the source node’s sensor nodes to the destination node as an example, the source node transmits data to the destination node in a multi-hop manner up until the network connectivity rate Lc, which is the point at which the number of nodes connected to the original source node no longer changes. The calculating Formula (18) is as follows:

(18) $$L_{c}=N_{1}/n$$

**Figure 8:**
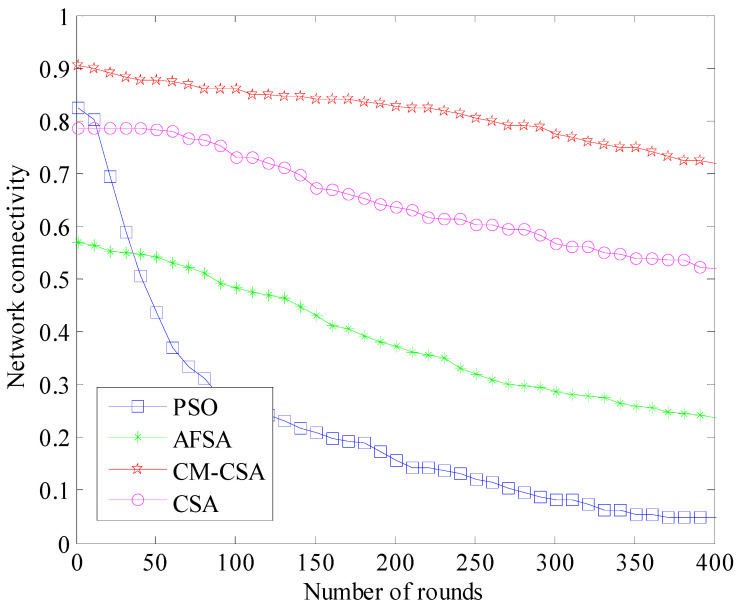
Comparison of network connectivity.

The number of nodes that can be sensed by a sensor node is *N*_1_, and the parameter *n* is the total number of sensor nodes in the entire sensor network. A comparison of the connectivity rate results of the four algorithms is shown in Figure 8.

According to Figure 8’s comparison of the four WSNs’ clustering data-collecting techniques’ connection performance, each algorithm’s network connectivity steadily declines as the number of simulated rounds rises. This is mostly due to the fact that as the simulation goes on, the network’s remaining energy steadily depletes, which causes a steady fall in the connection performance of the network. The PSO algorithm has the weakest network connectivity when the four algorithms’ network connectivity performance is compared. As the simulation duration lengthens, the connectedness of the other three networks steadily declines. The CSA algorithm has the smallest drop of the group, whereas the AFSA algorithm has the biggest fall. The CM-CSA method put forward in this study has a network connectivity drop that is always less than 0.85. The PSO method, AFSA algorithm, and basic CSA algorithm all have average network connectivity values of 0.4, 0.53, and 0.65, respectively. The suggested CM-CSA enhances the network connection performance by 52.9%, 37.6%, and 23.5% when compared to the PSO method, AFSA algorithm, and basic CSA algorithm. Overall, the connection performance of the CM-CSA algorithm network presented in this research is the best.

(8) Network reliability comparison

An important metric of network performance is the network’s dependability [32,33]. The connection *C*_1_ between the end nodes, the network connectivity rate *C*_2_, and the network capacity *C*_3_ are three factors that may be used to assess the dependability of *Rs*. Formula (19) equals [34]:

*R_s_* = 0.167*C*_1_ + 0.5*C*_2_ + 0.333*C*_3_ (19)

**Figure 9:**
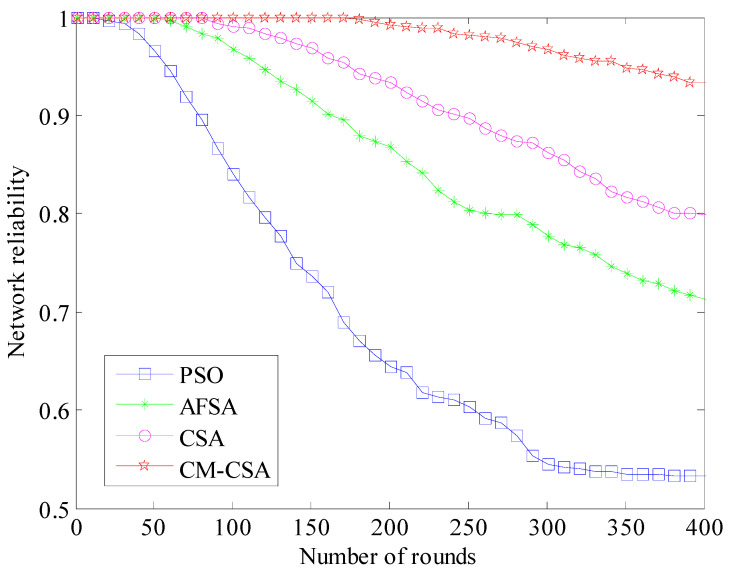
Comparison of network reliability.

The interconnection of end-to-end nodes is referred to as the dependability of network node connections. The reliability matrix is typically constructed using the distance between nodes as seen by WSNs, and the results are compared to random edge reliability matrix samples [35]. The average node connection dependability value after 50 rounds is found using the Monte Carlo methodology. The network survival probability is often calculated as the ratio of surviving nodes to the total number of network nodes [36]. The connection *C*_1_ between all nodes may be computed after 400 cycles using Monte Carlo analysis and the reliability of *Rs*, which is determined by the distance between the sensor nodes [37]. The ratio of the number of all surviving nodes to the total number of nodes in the network determines the value of the network capacity *C*_3_, which measures the survival probability of all nodes. The reliability values of the four algorithms are calculated, and the reliability comparison is shown in Figure 9.

Figure 9 shows that the network dependability is based on 0.9 after 300 cycles. The PSO algorithm’s network dependability has decreased dramatically by 40%, while the other three methods’ reliability has decreased by 30%. Only 5.7% of the suggested CM-CSA algorithm’s network dependability is lost. After 70 rounds, the PSO algorithm’s reliability reduces to 0.9, the AFSA algorithm’s reliability decreases to 0.9 after 150 rounds, and the CSA algorithm’s reliability drops to 0.9 after 250 rounds. After 400 rounds, the suggested CMCSA algorithm’s reliability is above 0.9, and the system stability is still greater than 0.9. The suggested clustered CM-CSA data-gathering technique offers the highest data transmission process reliability.

(9) Comparison of network load balance

**Figure 10:**
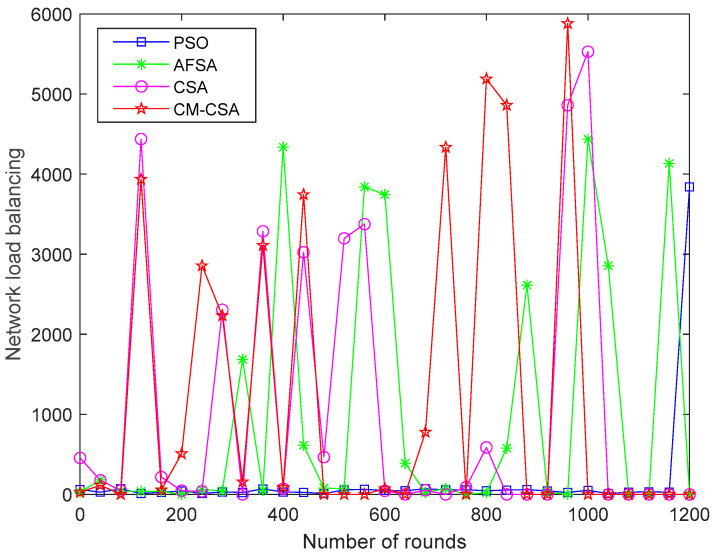
Comparison of network load balance.

Refer to [38,39,40] for the calculation algorithm for network load balancing. We can determine the four techniques’ network load balancing performance using the formula in references [38,39]. The simulation results of a network load balance comparison are shown in Figure 10.

The better the network balance, the higher the network load balance value. According to a comparison of the four algorithms, the PSO method has a poor network balance, whereas the AFSA strategy, the CSA algorithm, and the CM-CSA algorithm suggested in this research have similar network load balances and perform rather well in terms of network load balancing.

The CM-CSA method suggested in this research is the best in convergence speed and the best in finding the optimal solution compared with PSO algorithm, AFSA algorithm, and basic CSA algorithm, according to the results of eight test functions and the optimization results of wireless sensor network topology. However, the performance of the algorithm optimization is not steady or good at first. The proposed algorithm’s simulation duration is comparatively more prolonged.

## 8. Conclusions

The performance of the routing algorithm, a crucial area of study for wireless sensor networks, has a direct impact on the network’s lifespan. This research suggests an ideal clustering approach for wireless sensor networks that enhances the Cauchy mutation crow algorithm based on an examination of existing clustering routing methods. The range of variation and the global search capabilities can be increased with Cauchy mutation to promote population variety and prevent settling into the local optimum. The discriminative probability should be introduced, and when the leader realizes he is being followed, the current person’s location update approach should be improved. The suggested algorithm balances the network nodes’ energy usage, boosts productivity, and significantly extends the network’s lifespan.

The cluster head selection mechanism will then be further optimized, and it will be combined with other recently created clustering protocols to investigate novel cluster head selection methods. Simultaneously, the performance of the suggested method is further enhanced by numerous tests and applications to increasingly expand WSNs’ applications. Another issue worth researching regarding the WSN cluster routing algorithm is how the cluster head may execute data fusion more effectively in each cycle of the low-power cluster routing algorithm.

## Figures and Tables

**Table 1:** The procedures for implementing the CM-CSA algorithm.

Input: The Initial Position of the Crow Population Output: The Optimal Solution of the Problem (the Best Position in the Memory of All Crows) and the Optimal Individual
Step 1: Start
Step 2: Initialization. Randomly generate *n* crows in the *d*-dimensional feasible region, where each crow $X^{i,iter}=x_{1},x_{2},x_{3},\cdots ,x_{n}$ represents a feasible solution, and initialize the maximum iteration evaluation times and the awareness probability *AP*. Step 3: Initialize the crow memory value and calculate the fitness value.
Step 4: Update the parameter $g_{best}$.
Step 5: Use Formula (11) to perform Cauchy mutation on $g_{best}$ and perform cross-border processing.
Step 6: Use Formula (13) to adaptively adjust the step length *fl*.
Step 7: Use Formula (14) to perform displacement update operation.
Step 8: Check the feasibility of the new location.
Step 9: Calculate the fitness of the new position and update the memory value.
Step 10: Repeat steps 4 to 9 until the termination condition is reached, and then stop the iteration.
Step 11: Output the optimal solution and the optimal individual. Step 12: End.

**Table 2:** Implementation steps of clustering data collection for WSNs.

Step	Implementation Steps of Clustering Data Collection for WSNs
1	Step 1: Cluster data collection initialization, WSN system initialization. //Determine the location information of the node.
2	Step 2: Cluster head selection.
3	Calculate the average energy based on the information collected from all the nodes, and then set it as the energy threshold, and set the node with the remaining energy to be greater than the energy threshold as the candidate cluster head, and the cluster head will be selected from the candidate cluster heads.
4	Step 3: Cluster formation (calculation of the optimal number of cluster heads). //Based on the CM-CSA algorithm WSN optimal number of clusters.
5	Initialize the CM-CSA algorithm coefficients, including perception probability *AP* and flight step length *fl*, crow population location and crow memory.
6	Determine the current crow population’s objective function value.
7	The population is updated and corrected, and the crow memory is updated, using the multi-probability random walk approach.
8	Update the crow memory while calculating the objective function value of the current crow population.
9	Adopt an elite retention approach to keep top performers and systematically get rid of underperformers.
10	Update the perception probability *AP* and flight step length *fl*.
11	If the algorithm reaches the termination condition, the search stops and the optimal position of the crow is output, which is the optimal solution. Otherwise, return to step 6, and search for the optimal solution again.
12	Output the optimal number of cluster heads calculated by the CM-CSA algorithm for clustering.
13	Step 4: Data transfer between clusters.
14	Step 5: Stable operation of the network.
15	End

**Table 3:** The test functions.

Function	Equation	Dimension	Bounds	Optimum
F1	${\sum_{i=1}^{d}\left(\sum_{j=1}^{i}x_{j}\right)}^{2}$	30	[−100,100]	0
F2	$\max\left\{\left|x_{i}\right|,1\leq i\leq d\right\}$	30	[−100,100]	0
F3	$\sum_{i=1}^{d-1}\left[100{\left(x_{i+1}-x_{i}^{2}\right)}^{2}+{\left(x_{i}-1\right)}^{2}\right]$	30	[−10,100]	0
F4	$\sum_{i=1}^{d}{\left(\left|x_{i}+0.5\right|\right)}^{2}$	30	[−100,100]	0
F5	$\sum_{i=1}^{d}ix_{i}^{4}+rand(0,1)$	30	[−1.28,1.28]	0
F6	$\sum_{i=1}^{d}-x_{i}\sin(\sqrt{\left|x_{i}\right|})$	30	[−500,500]	0
F7	$10d+\sum_{i=1}^{d}\left[x_{i}^{2}-10\cos(2\pi x_{i})\right]$	30	[−100,100]	0
F8	$-20\exp\left(-0.2\sqrt{\frac{1}{d}\sum_{i=1}^{d}x^{2}}\right)-\exp\left(\frac{1}{d}\sum_{i=1}^{d}\cos(2\pi x_{i})\right)+20+\exp(1)$	30	[−5.12,5.12]	0
F9	$\frac{1}{4000}\sum_{i=1}^{d}x_{i}^{2}-\prod_{i=1}^{d}\cos\frac{x_{i}}{\sqrt{i}}+1$	30	[−32,32]	0
F10	${\left(\frac{1}{500}+\sum_{j=1}^{25}\frac{1}{j+\sum_{i=1}^{2}{\left(x_{i}-a_{ij}\right)}^{6}}\right)}^{-1}$	30	[−65,65]	0

**Table 4:** Test results of CEC functions.

Function	Algorithm	Mean	Std	Best
F1	PSO	65.066373	12.231686	38.688703
	FA	517.992251	1526.295420	9.142594
	SCA	7283.128602	7731.478783	458.277701
	AFSA	4.96 × 10^4^	1.67 × 10^5^	7.63 × 10^3^
	CSA	1.27 × 10^3^	5.50 × 10^3^	1.47 × 10^−6^
	CM-CSA	1.02 × 10^2^	9.31 × 10^2^	3.36 × 10^−7^
F2	PSO	1.559970	0.119099	1.309212
	FA	2.684358	0.597503	1.780876
	SCA	2.602636	1.178328	0.791869
	AFSA	3.61 × 10	1.28 × 10^−1^	2.02 × 10
	CSA	1.21	2.11	1.35 × 10^−2^
	CM-CSA	5.03 × 10	2.67 × 10^2^	1.29 × 10^−7^
F3	PSO	3180.719584	791.918414	1794.991035
	FA	3511.842488	2686.516869	846.406105
	SCA	6830.271763	12,443.989131	33.533474
	AFSA	2.64 × 10^3^	1.18 × 10^4^	3.52 × 10^2^
	CSA	3.47 × 10	2.18 × 10	2.50 × 10^−1^
	CM-CSA	3.15 × 10	1.86 × 10	2.56 × 10^−2^
F4	PSO	1.43 × 10	1.95	8.09 × 10^−1^
	FA	6.27	1.7	3.16
	SCA	8.478817	8.899730	3.850588
	AFSA	1.16 × 10^3^	1.78 × 10^3^	2.73 × 10^3^
	CSA	3.37 × 10^−2^	1.10 × 10^−3^	2.04 × 10^−4^
	CM-CSA	1.47 × 10^−1^	7.09 × 10^−3^	2.88 × 10^−5^
F5	PSO	95.000827	14.253399	60.619186
	FA	8.869441	2.482767	3.959825
	SCA	0.044490	0.032707	0.006100
	AFSA	1.15	2.99 × 10	1.48
	CSA	9.39 × 10^−2^	2.07 × 10^−1^	2.39 × 10^−2^
	CM-CSA	2.50 × 10^−2^	6.86 × 10^−3^	2.28 × 10^−4^
F6	PSO	311.245852	97.179060	97.579370
	FA	219.176033	129.775277	59.693352
	SCA	2021.5515	4320.47	268.504861
	AFSA	6.66	4.02 × 10^−1^	1.37 × 10^−2^
	CSA	2.73 × 10^−1^	2.85 × 10^−1^	1.74 × 10^−3^
	CM-CSA	3.02 × 10^−2^	2.27 × 10^−2^	4.48 × 10^−4^
F7	PSO	3.87 × 10	5.81 × 10	4.14 × 10
	FA	1.50 × 10^2^	3.23 × 10	8.54 × 10^−2^
	SCA	5.47 × 10	4.75 × 10^−1^	4.15 × 10^−2^
	AFSA	8.54	5.14 × 10^−1^	8.11 × 10^−2^
	CSA	2.16 × 10^−1^	2.87 × 10^−1^	1.87 × 10^−2^
	CM-CSA	4.56 × 10^−2^	2.85 × 10^−2^	8.67 × 10^−3^
F8	PSO	7.42 × 10	9.57	5.71 × 10^−1^
	FA	3.68	6.76 × 10^−1^	4.91 × 10^−2^
	SCA	5.51 × 10	7.36 × 10^−1^	9.54 × 10^−2^
	AFSA	6.58 × 10	4.02 × 10^−1^	1.37 × 10^−2^
	CSA	2.73 × 10^−1^	2.85 × 10^−1^	1.98 × 10^−3^
	CM-CSA	3.02 × 10^−2^	2.27 × 10^−2^	5.17 × 10^−4^
F9	PSO	4.98 × 10^2^	5.12 × 10^2^	2.17 × 10^2^
	FA	3.67 × 10^2^	5.49 × 10^−1^	4.82 × 10^−2^
	SCA	8.98 × 10	7.49 × 10^−1^	6.57 × 10^−1^
	AFSA	5.69	3.14 × 10^−1^	3.98 × 10^−1^
	CSA	4.61 × 10^−1^	2.49 × 10^−1^	2.69 × 10^−2^
	CM-CSA	4.28 × 10^−2^	3.57 × 10^−1^	6.81 × 10^−7^
F10	PSO	7.84 × 10^2^	2.97 × 10^2^	8.54 × 10
	FA	2.61 × 10	5.42 × 10^−1^	3.64 × 10^−2^
	SCA	5.24 × 10^3^	3.67 × 10	1.47 × 10
	AFSA	6.21 × 10	6.51 × 10^−1^	2.97 × 10^−1^
	CSA	2.64 × 10^−1^	2.85 × 10^−1^	4.65 × 10^−2^
	CM-CSA	5.68 × 10^−1^	4.32 × 10^−2^	2.59 × 10^−5^

## Data Availability

The data used to support the findings of this study are available from the corresponding author upon request.
